# Mechanistic insights into inositol-mediated rumen function promotion and metabolic alteration using *in vitro* and *in vivo* models

**DOI:** 10.3389/fvets.2024.1359234

**Published:** 2024-02-16

**Authors:** Guopei Yin, Zhe Sun, Zhanqing Wang, Yuanhong Xia, Long Cheng, Guixin Qin, Natnael D. Aschalew, Hongyun Liu, Xuefeng Zhang, Qilu Wu, Weigang Zhang, Wei Zhao, Tao Wang, Yuguo Zhen

**Affiliations:** ^1^Key Laboratory of Animal Nutrition and Feed Science of Jilin Province, Key Laboratory of Animal Production Product Quality and Security Ministry of Education, JLAU-Borui Dairy Science and Technology R&D Center, College of Animal Science and Technology, Jilin Agricultural University, Changchun, China; ^2^College of Life Sciences, Engineering Research Center of Bioreactor and Pharmaceutical Development, Ministry of Education, Jilin Agricultural University, Changchun, China; ^3^Postdoctoral Scientific Research Workstation, Feed Engineering Technology Research Center of Jilin Province, Changchun Borui Science and Technology Co., Ltd., Changchun, China; ^4^College of Agriculture and Environmental Science, Dilla University, Dila, Ethiopia; ^5^College of Animal Sciences, Zhejiang University, Hangzhou, China

**Keywords:** inositol, sheep, rumen fermentation, rumen microorganisms, metabolomics

## Abstract

Inositol is a bioactive factor that is widely found in nature; however, there are few studies on its use in ruminant nutrition. This study investigated the effects of different inositol doses and fermentation times on rumen fermentation and microbial diversity, as well as the levels of rumen and blood metabolites in sheep. Rumen fermentation parameters, microbial diversity, and metabolites after different inositol doses were determined *in vitro*. According to the *in vitro* results, six small-tailed Han sheep fitted with permanent rumen fistulas were used in a 3 × 3 Latin square feeding experiment where inositol was injected into the rumen twice a day and rumen fluid and blood samples were collected. The *in vitro* results showed that inositol could increase *in vitro* dry matter digestibility, *in vitro* crude protein digestibility, NH_3_-N, acetic acid, propionic acid, and rumen microbial diversity and affect rumen metabolic pathways (*p* < 0.05). The feeding experiment results showed that inositol increased the blood concentration of high-density lipoprotein and IgG, IgM, and IL-4 levels. The rumen microbial composition was significantly affected (*p* < 0.05). Differential metabolites in the rumen were mainly involved in ABC transporters, biotin metabolism, and phenylalanine metabolism, whereas those in the blood were mainly involved in arginine biosynthesis and glutathione and tyrosine metabolism. In conclusion, inositol improves rumen function, affects rumen microorganisms and rumen and blood metabolites and may reduce inflammation, improving animal health.

## 1 Introduction

Research on ruminant feed nutrition can improve in livestock production (1–4). The rumen is a unique digestive organ that contains many microorganisms living in symbiosis with the host. These bacteria, which are involved in the decomposition and utilization of feeds, produce corresponding metabolites that further promote the growth of the host (5). This complex internal environment makes the nutritional regulation of ruminants difficult; therefore, an environmentally friendly and non-harmful feed additive is imperative to improve rumen fermentation capacity, regulate rumen microorganisms and metabolites, enhance feed-utilization efficiency of ruminants and boost the economic benefits of the farms.

Inositol is a cyclic sugar alcohol widely abundant in fresh fruits, vegetables, and cereals. It is involved in the metabolic activities of cells (6) and is regarded as an active substance essential for the growth of living organisms (7). Our group has previously determined that inositol is a potentially potent metabolite in yeast cultures and is involved in glycine, serine, and threonine metabolic pathways (8). Inositol has many biological functions and plays essential roles in cellular signaling, intracellular transport, and osmoregulation (9, 10). Cellular deficiency of inositol affects phospholipid metabolism and related gene expression and causes cell death (11), demonstrating its essential role in the maintenance of regular cellular activity and metabolic functions. Inositol is often used as a feed additive for aquatic animal production, where it promotes growth and enhances antioxidant capacity (12, 13); however, despite numerous studies in different fields, few have investigated the effects of inositol in ruminants. In this study, the effects of inositol on rumen fermentation function, rumen microbial diversity, blood indices, and rumen and blood metabolites in sheep were investigated. This study provides a reference for the application of inositol in ruminant production.

## 2 Materials and methods

### 2.1 Animal ethics

The experiments were conducted at the College of Animal Science and Technology, Jilin Agricultural University. All experimental procedures were performed by the Guidelines for the Care and Use of Experimental Animals of the Jilin Agricultural University (JLAU-ACUC2022-006).

### 2.2 Experimental design

**Figure 1:**
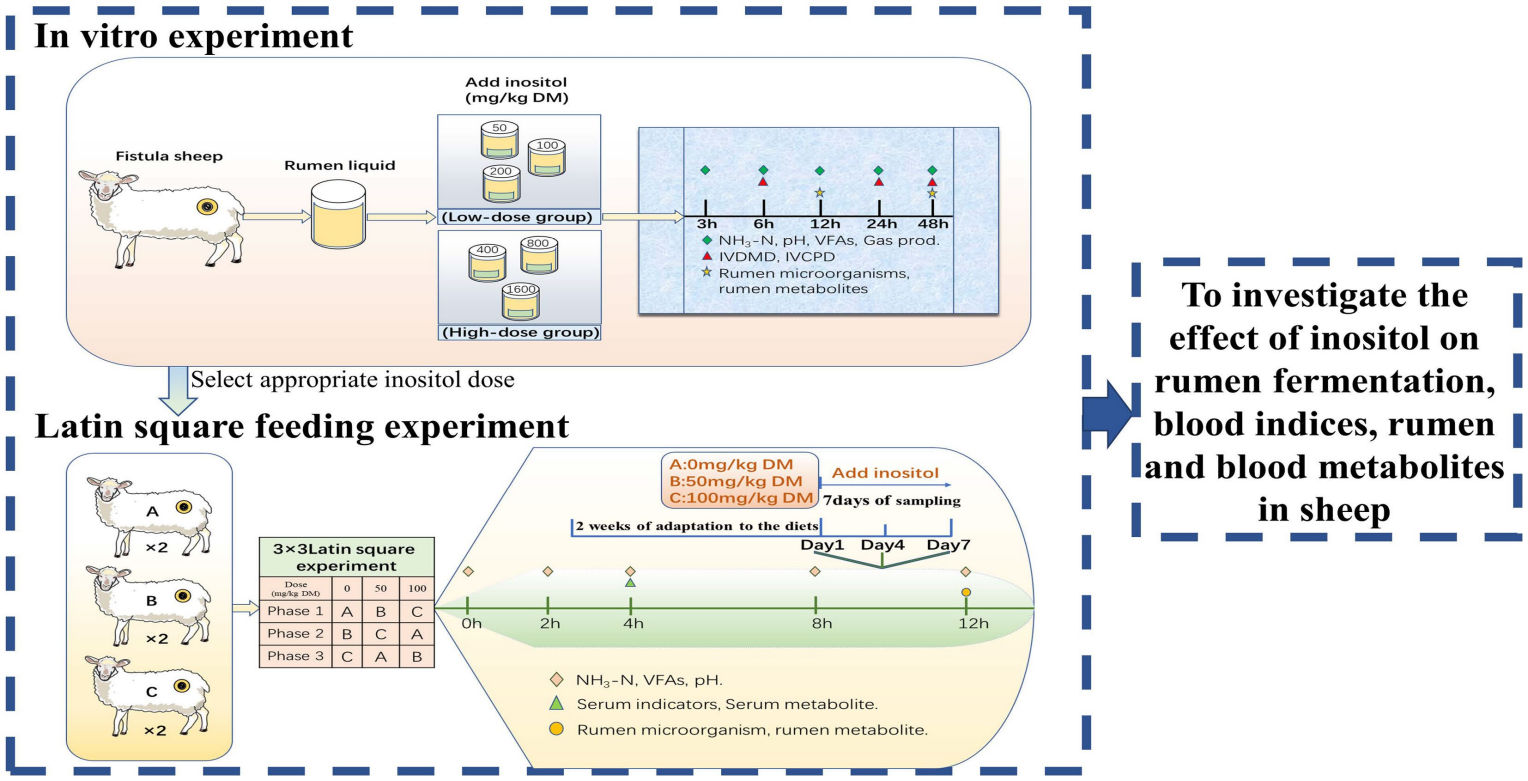
Experimental design.

The experiment involved an *in vitro* and a Latin square feeding experiment. An appropriate dose of inositol was selected for the feeding experiment based on the *in vitro* results. The experimental results were combined to preliminarily investigate the effects of inositol on rumen function, blood indices, and metabolites (Figure 1).

*In vitro* experiments were designed based on two factors. Factor1 was supplemental inositol dose (Feed grade inositol, 99%; Anhui Yuanzheng Biological, Anhui, China). The experiment included control (0 mg/kg DM), low-dose inositol-supplemented (50, 100, or 200 mg/kg DM), and high-dose inositol-supplemented (400, 800, or 1,600 mg/kg DM) groups. Factor 2 was fermentation time (3, 6, 12, 24, or 48 h). There were 12 replicates in each group (four replicates per experiment; the experiment was repeated three times).

The 3 × 3 Latin square experiment used six experimental animals divided into three groups, 0, 50, or 100 mg/kg DM inositol, with two replicates per group. Each experimental period consisted of 21 days, divided into 14 days for adaptation to the diets and 7 days for sampling. During the sampling period, inositol was injected into the rumen using a disposable syringe every day before feeding. Inositol was diluted with ultra-pure water in advance to the required concentration. The samples were collected on days 1, 4, and 7 for further analysis.

Male small-tail Han sheep (40 ± 1.5 kg) were kept in individual cages and fed twice daily (08:00 and 20:00) at a ratio of 4:6 concentrate (pellet feed; Table 1) to roughage (oat hay), according to the feeding standard of Feed standard of meat-producing sheep and goats (NY/T816-2004). The content of inositol in the diet had been tested by a professional testing company before the experiment, and no inositol content was detected in all the diets.

**Table 1:** Ingredients and nutritional composition of pellet feed (dry matter basis, %).

Item	Content
Ingredients	%
Corn	37.0
Brown rice mixture	6.0
Soybean meal	28.5
Cotton meal	1.0
Sesame meal	3.0
Distillers dried grains with soluble	6.0
Spouting corn bran	6.0
Corn bran	6.0
Bentonite	1.0
Molasses	1.5
Premix^1^	4.0
Amount	100
Nutritional composition	%
Metabolic energy (MJ/kg)^2^	11.3
Crude protein	23.8
Neutral detergent fiber	15.0
Acid detergent fiber	7.2
Ether extract	3.9
Ash	11.4
Sugar	6.5
Starch	35.9

1 Premix composition (per kilogram): FeSO_4_. 179 mg; CuSO_4_•5H_2_O. 23 mg; ZnSO_4_•5H_2_O. 92 mg; MnSO_4_. 70 mg; Vitamin A. 16000 IU; Vitamin D. 111000 IU; Vitamin E. 915 IU.

2 ME was calculated according to NRC (2001).

### 2.3 *In vitro* experiment

After multi-layer gauze filtration, the rumen fluid was mixed with buffer at a ratio of 1:2 and placed into a fermentation flask containing substrate and inositol. Once the mixture was homogeneous, carbon dioxide was continuously injected into the vessel, and the vessel was placed in a gas-bath oscillator bed for a constant-temperature oscillatory incubation (39°C, 80 rpm). The amount of gas production was recorded with the ANKOM^RF^ gas production system (ANKOM Technology, Macedon, NY, United States). Buffer configuration was based on the method described by Menke and Steingass (14).

### 2.4 Sample collection

A storage container was pre-warmed and filled with CO_2_ to ensure an anaerobic environment for the survival of rumen microorganisms. Rumen fluid was collected from different parts of the rumen of sheep with permanent rumen fistulas on the day of the experiment and mixed before being returned to the laboratory and filtered with multi-layer gauze. During this period, the external ambient temperature (39–41°C) of the rumen fluid was maintained, and CO_2_ was continuously injected. Blood was collected from the neck vein into a coagulant tube. The supernatant was collected by centrifugation at 3,000 × g at 4°C for 10 min and stored at −80°C for later use.

### 2.5 Determination of rumen fermentation parameters

The pH was measured by a Sanxin MP523-04 portable pH meter (Shanghai Sanxin Instrument Co., Ltd., Shanghai, China). The concentration of ammonia nitrogen (NH_3_-N) was determined by colourimetric analysis using an ultraviolet spectrophotometer (UV-1201; Shimadzu, Tokyo, Japan). The VFAs were determined by meteorological chromatography (Agilent 7890B, Agilent Technologies, Santa Clara, CA, United States). Total gas production during *in vitro* fermentation was measured using an ANKOM^RF^ gas generator and GPM software (ANKOM Technology). The formula for calculating gas production was where *V_y_* is the volume of gas produced in mL; *V_x_* represents the volume of gas in the upper space inside each sample bottle in mL; Ppsi is the cumulative pressure at a certain time of fermentation, expressed in psi. After *in vitro* fermentation, the substrate was dried at 105°C until a constant weight was achieved, and the *in vitro* dry matter digestibility (IVDMD) was calculated. *In vitro* crude protein digestibility (IVCPD) was calculated by mixing the same fermentation substrate and determining the crude protein (15).

### 2.6 Blood index determination

Total protein, albumin, globulin, total cholesterol, blood glucose, high-density lipoprotein, low-density lipoprotein, triglyceride, urea, free fatty acid, and β-hydroxybutyrate, were determined using a Mindray BS-400 automatic blood biochemical analyzer (Shenzhen, China). Insulin levels were determined using the Insulin Enzyme-Linked Immunoassay Kit (HY-D0001; Beijing Huaying Biological, Beijing, China).

Sheep immunoglobulin A (IgA), Sheep Immunoglobulin G (IgG), Sheep Immunoglobulin M (IgM), and the levels of sheep IL-2, IL-4, IL-6, IL-10, soluble leukocyte differentiation antigen 14 (sCD14), and TNF-α were determined using reagents provided by Shanghai Jining Biological Institute Box. The T-AOC, total oxidant status, total antioxidant status (TAS), GSH-Px, MDA, and SOD were determined using kits provided by the Nanjing Jiancheng Institute of Biotechnology, according to the manufacturer’s instructions.

### 2.7 Determination of rumen microbial diversity

On days 1, 4, and 7, sheep rumen fluid was collected 12 h after morning feeding for microbiological analysis. Total DNA from rumen microorganisms was extracted using an E.Z.N.A. Soil DNA Kit (D5625-01; Omega Bio-Tek, Norcross, GA, United States), and nucleic acids were quantified using an ultraviolet spectrophotometer. The highly variable V3–V4 region of the bacterial 16S rDNA gene with a length of about 468 bp was selected for sequencing. The V3–V4 region-specific primers F: ACTCCTACGGGAGGCAGCA and R: GGACTACHVGGGTWTCTAAT were selected for PCR amplification. Each primer contained a 7 bp oligonucleotide sequence to distinguish different samples from the same library. Q5 DNA high-fidelity polymerase was used for the PCR (New England Biolabs, Ipswich, MA, United States). The PCR products were quantified on an FLx800 microplate reader (BioTek, Winooski, VT, United States) using the Quant-iT PicoGreen dsDNA Assay Kit, Quantification of the PCR products was performed on an FLx800 microplate reader (BioTek, Winooski, VT, United States) using the Quant-iT PicoGreen dsDNA Assay Kit, and the samples were then normalized. An Illumina TruSeq Nano DNA LT Library Prep Kit (San Diego, CA, USA) was used for library preparation, and an Agilent Sensitivity DNA Kit was used to conduct library quality inspection on an Agilent Bioanalyzer. Paired-end sequencing of the libraries was then performed on an Illumina NovaSeq using the NovaSeq 6000 SP Reagent Kit (500 cycles). The DADA2 plug-in was used to remove primer sequences, filter, denoise, and remove chimeric sequences from the raw data (16).

### 2.8 Determination of rumen metabolites

One hundred microliters of rumen fluid was placed in a 2 mL centrifuge tube and 400 μL of pre-cooled methanol was added. The samples were vortexed for 30 s and sonicated in a water bath for 15 min, after which they were centrifuged at 13,800 × g for 15 min at 4°C. The supernatants were then concentrated and dried in a lyophilizer. Thirty microlitres of methylammonium salt reagent (1 mL of pyridine per 20 mg of methylammonium hydrochloride) were added to the concentrated samples, which were gently shaken and mixed well before incubating at 80°C for 30 min. Forty microlitres of BSTFA (containing 1% TMCS, v/v, REGIS Technologies) was then added, and the samples were incubated for 1.5 h at 70°C. After incubation, the samples were cooled down to 25°C, and 5 μL of saturated fatty acid methyl ester (FAMEs, dissolved in chloroform) was added. The samples were then tested by gas chromatography (7890B, Agilent) coupled with a time-of-flight mass spectrometer (PEGASUS HT, LECO, GC-TOF-MS).

### 2.9 Determination of blood metabolites

One hundred microliters of blood sample were placed in a 1.5 mL centrifuge tube, 400 μL of extraction solution (methanol: acetonitrile = 1:1 (V/V), including internal standard) was then added, the sample vortexed for 30 s, sonicated for 10 min in an ice-water bath, and incubated for 1 h at −40°C to precipitate proteins, then centrifuged at 13,800 × g for 15 min at 4°C. The supernatant was carefully withdrawn into a feed bottle. The quality control (QC) sample was prepared by mixing an equal aliquot of the supernatants from all of the samples. LC–MS/MS analyses were performed using a UHPLC system (Vanquish, Thermo Fisher Scientific) with a UPLC BEH Amide column (2.1 mm × 100 mm, 1.7 μm) coupled to Orbitrap Exploris 120 mass spectrometer (Orbitrap MS, Thermo) (17). The mobile phase consisted of 25 mmol/L ammonium acetate and 25 mmol/L ammonium hydroxide in water (pH = 9.75) (A) and acetonitrile (B). The auto-sampler temperature was 4°C, and the injection volume was 2 μL.

### 2.10 Statistical analysis

The results were analyzed using a general linear model for two-factor trials with IBM SPSS Statistics 23 software (IBM Corp., Armonk, NY, United States). Duncan’s multiple comparisons were used to test the level of significance of factors 1 and 2 and their interactions (Factor 1 × Factor 2). *p*-values with *p* < 0.05 were considered significant differences, and those with *p* < 0.01 were considered highly significant differences.

The rumen metabolome data were analyzed using ChromaTOF v 4.3 software (LECO Corp., St. Joseph, MI, United States) for peak extraction, baseline correction, deconvolution, peak integration, and peak alignment of the mass spectrometry data. The LECO-Fiehn Rtx5 database was used for the substance characterization, including mass spectrum and retention time index matching. Peaks detected in less than half of QC samples or RSD > 30% in QC samples were removed (18).

After the raw data of blood metabolites were converted into mzXML format by ProteoWizard software (Palo Alto, CA, United States), the identified peak maps were processed (peak identification, peak extraction, peak alignment, and integration) using an in-house written R language package with kernel XMCS, and then matched with the secondary mass spectrometry database for substance annotation. The Cutoff value for algorithm scoring was set to 0.3.

## 3 Results

### 3.1 Rumen fermentation parameters *in vitro*

The results showed that different inositol doses and fermentation times had highly significant effects on IVDMD and IVCPD (*p* < 0.01). Gas production, NH_3_-N, pH, and concentrations of total volatile fatty acid (TVFA), acetic acid, isobutyric acid, butyric acid, isovaleric acid, and valeric acid were significantly affected by the reciprocal effect between the inositol dose and the fermentation time (*p* < 0.01; Table 2). The low-dose inositol groups improved rumen fermentation function, promoted the digestion of nutrients, and increased acid production during rumen fermentation relative to other experimental groups.

**Table 2:** *In vitro* rumen fermentation parameters at different inositol doses^1^.

		Dose (Inositol mg/kg DM)								*p*-value		
Items	Time (h)	0	50	100	200	400	800	1,600	Sem	Dose	Time	D × T
IVDMD, %	6	24.27^c^	38.66^a^	33.01^ab^	31.94^b^	17.55^d^	17.42^d^	20.11^cd^	0.01	<0.01	<0.01	0.93
	24	37.76^ab^	45.23^a^	44.49^a^	40.08^ab^	31.41^cd^	29.4^d^	32.6^bcd^	0.01			
	48	44.59^bc^	57.56^a^	54.77^a^	50.18^ab^	37.24^c^	38.28^c^	40.75^c^	0.01			
IVCPD, %	6	18^cd^	26.04^a^	19.15^bcd^	25^ab^	14.55^d^	19.49^bcd^	23.22^abc^	0.009	<0.01	<0.01	<0.01
	24	36.95^a^	32.03^ab^	37.08^a^	31.53^b^	25.94^b^	32.42^ab^	29.6^ab^	0.009			
	48	45.33^a^	49.42^a^	52.72^a^	52.6^a^	32.84^b^	36.76^b^	32.77^b^	0.011			
Gas PROD, mL	3	43.81^ab^	47.41^ab^	35.23^bc^	49.57^a^	43.49^ab^	24.93^c^	34.81^bc^	1.82	<0.01	<0.01	<0.01
	6	84.02^ab^	78.25^abc^	71.08^bc^	75.31^bc^	94.45^a^	50.34^d^	61.78^cd^	2.49			
	12	126.6^ab^	149.91^a^	110.73^b^	122.37^ab^	147.88^a^	73.91^c^	106.44^b^	4.3			
	24	134.9^cd^	246.66^a^	174.61^bc^	188.98^b^	200.71^b^	127.59^d^	124^d^	6.99			
	48	192.36^bc^	312.59^a^	259.39^ab^	257.35^ab^	272.65^b^	147.04^d^	142.7^d^	11.33			
NH_3_-N, mg/dL	3	16.14^a^	16.69^a^	18.02^a^	16.3^a^	13.06^b^	10.9^b^	12.27^b^	0.47	<0.01	<0.01	<0.01
	6	19.14^a^	19.9^a^	19.89^a^	16.77^a^	13.22^b^	12.55^b^	11.77^b^	0.58			
	12	27.28^a^	26.32^a^	28.12^a^	25.5^a^	17.67^b^	14.5^b^	13.9^b^	0.94			
	24	36.35^a^	31.48^ab^	30.89^abc^	35.56^a^	22.92^c^	25.78^bc^	26.61^bc^	1.11			
	48	65.1^a^	65.37^a^	63.12^a^	63.53^a^	41.24^b^	41.58^b^	44.53^b^	1.91			
pH value	3	7.19^ab^	6.97^cd^	6.88^d^	7.07^bc^	7.09^bc^	7.25^a^	7.23^a^	0.02	<0.01	<0.01	<0.01
	6	7.13^ab^	6.86^c^	6.80^c^	7.04^b^	7.09^b^	7.21^a^	7.19^a^	0.02			
	12	7.09^ab^	6.58^c^	6.61^c^	6.98^b^	6.98^b^	7.18^a^	7.13^a^	0.03			
	24	6.29^b^	6.39^b^	6.47^b^	6.26^b^	6.86^a^	6.79^a^	6.81^a^	0.04			
	48	6.22^b^	6.25^b^	6.27^b^	6.14^b^	6.34^ab^	6.52^a^	6.53^a^	0.03			
TVFA, mmol/L	3	24.70^bc^	38.91^a^	41.19^a^	29.61^b^	29.33^b^	17.94^c^	21.84^c^	1.24	<0.01	<0.01	<0.01
	6	33.06^cd^	42.65^abc^	48.65^a^	46.01^ab^	37.89^bc^	22.37^e^	25.71^de^	1.58			
	12	37.96^c^	59.15^ab^	65.11^a^	64.01^ab^	52.97^b^	30.67^c^	35.96^c^	2.06			
	24	65.08^b^	85.17^a^	79.45^a^	74.70^b^	69.82^a^	73.87^a^	77.59^a^	4.2			
	48	120.1^bc^	126.18^bc^	125.41^bc^	115.21^c^	177.58^a^	154.18^ab^	137.09^bc^	4.63			
Acetate acid, mmol/L	3	14.85^cd^	19.67^ab^	21.09^a^	15.34^c^	16.67^bc^	11.66^d^	13.81^cd^	0.55	<0.01	<0.01	<0.01
	6	19.53^bc^	21.22^ab^	24.34^a^	22.67^ab^	20.88^ab^	14.33^d^	16.25^cd^	0.66			
	12	22.51^b^	28.68^a^	31.24^a^	32.06^a^	29.41^a^	18.96^b^	21.76^b^	0.87			
	24	32.32^b^	50.54^a^	48.47^a^	34.85^b^	44.29^a^	45.53^a^	47.70^a^	1.24			
	48	61.57^cd^	63.6^cd^	63.39^cd^	55.95^d^	97.76^a^	87.22^ab^	77.63^bc^	2.64			
Propionate acid, mmol/L	3	5.46^cd^	11.86^a^	12.62^a^	8.53^b^	6.57^bc^	3.69^d^	4.92^cd^	0.48	<0.01	<0.01	<0.01
	6	7.61^bc^	13.21^a^	15.34^a^	14.57^a^	8.81^b^	4.87^c^	5.90^bc^	0.62			
	12	8.74^bc^	18.64^a^	21.22^a^	18.88^a^	11.85^b^	7.37^c^	9.00^bc^	0.79			
	24	18.81^ab^	21.20^ab^	18.64^ab^	23.69^a^	16.36^b^	16.78^b^	18.17^b^	0.66			
	48	32.00^b^	33.92^ab^	35.36^ab^	34.52^ab^	43.33^a^	36.46^ab^	32.30^b^	3.88			
Isobutyric acid, mmol/L	3	0.49^abc^	0.59^a^	0.61^a^	0.61^a^	0.57^ab^	0.41^c^	0.44^bc^	0.02	<0.01	<0.01	<0.01
	6	0.66^ab^	0.66^ab^	0.78^a^	0.79^a^	0.76^a^	0.48^b^	0.49^b^	0.03			
	12	0.75^bc^	1.00^ab^	1.14^a^	1.23^a^	1.09^ab^	0.60^c^	0.65^c^	0.05			
	24	1.00^b^	1.15^ab^	1.30^ab^	1.34^a^	1.24^ab^	1.37^a^	1.40^a^	0.04			
	48	1.96^b^	2.32^ab^	2.26^ab^	2.04^ab^	2.73^a^	2.35^ab^	2.23^ab^	0.08			
Butyric acid, mmol/L	3	2.78^cd^	4.85^a^	4.64^a^	3.42^bc^	4.10^ab^	1.40^e^	1.79^de^	0.2	<0.01	<0.01	<0.01
	6	3.70^b^	5.39^a^	5.40^a^	5.25^a^	5.47^a^	1.71^c^	2.05^c^	0.26			
	12	4.06^b^	7.36^a^	7.12^a^	7.76^a^	7.59^a^	2.41^b^	3.07^b^	0.33			
	24	8.09^a^	8.13^a^	7.24^ab^	8.30^a^	4.96^b^	6.19^ab^	6.33^ab^	0.32			
	48	14.75^b^	15.76^b^	13.83^b^	12.61^b^	21.16^a^	16.74^ab^	15.52^b^	0.69			
Isovaleric acid, mmol/L	3	0.74^ab^	0.87^a^	0.89^a^	0.85^a^	0.89^a^	0.55^c^	0.58^bc^	0.03	<0.01	<0.01	<0.01
	6	1.02^ab^	0.99^ab^	1.17^a^	1.16^a^	1.27^a^	0.68^b^	0.67^b^	0.05			
	12	1.21^bc^	1.68^ab^	1.83^a^	1.99^a^	1.97^a^	0.90^c^	0.95^c^	0.08			
	24	1.99	2.24	2.32	2.37	1.79	2.42	2.2	0.11			
	48	4.15^b^	4.92^ab^	4.38^ab^	3.74^b^	6.03^a^	5.17^ab^	4.55^ab^	0.22			
Valerate acid, mmol/L	3	0.40^d^	1.07^ab^	1.34^a^	0.85^bc^	0.52^cd^	0.23^d^	0.29^d^	0.06	<0.01	<0.01	<0.01
	6	0.53^c^	1.17^b^	1.63^a^	1.57^ab^	0.69^c^	0.30^c^	0.35^c^	0.08			
	12	0.69^c^	1.8^b^	2.57^a^	2.09^ab^	1.07^c^	0.44^c^	0.53^c^	0.12			
	24	2.86^b^	1.91^c^	1.48^c^	3.72^a^	1.19^c^	1.58^c^	1.79^c^	0.15			
	48	5.69	5.66	6.19	6.36	6.57	6.23	4.86	0.25			
A/P ratio	3	3.01^a^	1.73^b^	1.66^b^	2.23^b^	3.06^a^	3.20^a^	2.81^a^	0.1	<0.01	<0.01	<0.01
	6	2.82^a^	1.68^b^	1.67^b^	1.74^b^	2.89^a^	2.99^a^	2.76^a^	0.09			
	12	2.74^a^	1.63^b^	1.56^b^	1.84^b^	2.85^a^	2.65^a^	2.40^a^	0.08			
	24	0.85^c^	2.40^b^	2.63^a^	0.74^c^	2.72^a^	2.72^a^	2.64^a^	0.1			
	48	1.97^bc^	1.97^bc^	1.86^c^	1.63^c^	2.29^ab^	2.42^a^	2.49^a^	0.06			

Gas PROD = gas production; A/P ratio = acetate-to-propionate ratio; SEM = standard error of mean; Dose = different inositol doses; Time = *In vitro* rumen fermentation time; D × T = interaction of different inositol doses and *in vitro* rumen fermentation time.

1 0 mg/kg DM represents basal diet; 50 mg/kg DM, 100 mg/kg DM, 200 mg/kg DM, 400 mg/kg DM, 800 mg/kg DM and 1,600 mg/kg DM represent basal diet supplemented with 50, 100, 200, 400, 800, and 1,600 mg/kg DM inositol, respectively.

^a, b, c, d^ Means in the same row with different superscripts are significantly different (*p* < 0.05).

### 3.2 Microbial diversity *in vitro*

Based on the results of the *in vitro* rumen fermentation parameters, the low-dose inositol treatments were selected for rumen microbial diversity analysis at 12 and 48 h. A total of 3,813,300 original sequences were identified in all rumen samples, and 2,258,127 high-quality sequences were obtained.

**Figure 2:**
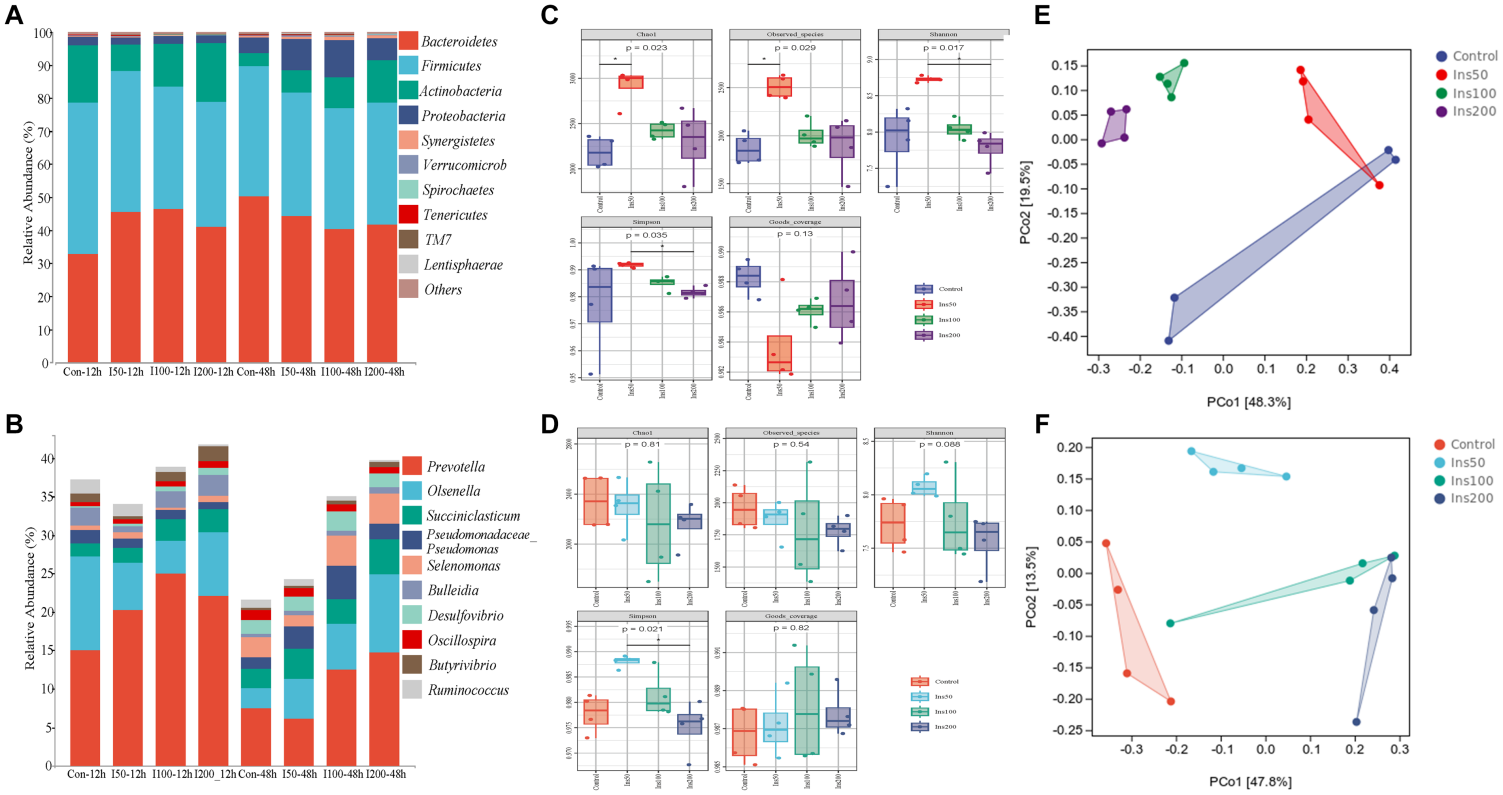
Effect of inositol supplementation on ruminal bacterial community *in vitro.* At the **(A)** phylum and **(B)** genus levels. Control-12 h, basal diet. Ins50-12 h, Ins100-12 h, and Ins200-12 h represent basal diet supplemented with 50, 100, and 200 mg/kg DM inositol, respectively, fermented *in vitro* for 12 h; Control-48 h, basal diet; Ins50-48 h, Ins100-48 h, and Ins200-48 h represent basal diet supplemented with 50, 100, and 200 mg/kg DM inositol, respectively, fermented *in vitro* for 48 h. The alpha diversity indices of the ruminal bacterial community. At **(C)** 12 and **(D)** 48 h. The beta diversity was estimated by principal coordinate analysis at **(E)** 12 and **(F)** 48 h. Control represents basal diet. Ins50, Ins100, and Ins200 represent basal diets supplemented with 50, 100, and 200 mg/kg DM inositol, respectively.

In all groups, the four most abundant phyla were Bacteroidetes, Firmicutes, Actinobacteria, and Proteobacteria, and the seven most abundant genera were *Prevotella*, *Olsenella*, *Succiniclasticum*, *Pseudomonadaceae_Pseudomonas*, *Selenomonas*, *Bulleidia*, and *Desulfovibrio* (Figures 2A,B). At the phylum level, different inositol doses significantly affected the abundance of Firmicutes and Spirochaetes (*p* < 0.05) and Proteobacteria, Tenericutes, and TM7 (*p* < 0.01). Different fermentation times significantly affected the abundance of Firmicutes, Actinobacteria, and TM7 (*p* < 0.05) and Proteobacteria, Synergistetes, Verrucomicrobia, Tenericutes, and Lentisphaerae (*p* < 0.01). The abundance of Bacteroidetes, Proteobacteria, and Synergistetes was significantly affected by the reciprocal effect between the inositol dose and fermentation time (*p* < 0.01). At the genus level, different inositol doses significantly affected the abundance of *Ruminococcus* (*p* < 0.05) and *Prevotella*, *Succiniclasticum*, and *Butyrivibrio* (*p* < 0.01). Different fermentation times significantly affected the abundance of *Prevotella*, *Succiniclasticum*, *Pseudomonadaceae_Pseudomonas*, *Selenomonas*, *Bulleidia*, *Desulfovibrio*, *Butyrivibrio*, and *Oscillospira* (*p* < 0.01). The abundance of *Selenomonas* and *Butyrivibrio* was significantly affected by the reciprocal effect between the inositol dose and fermentation time (*p* < 0.01).

The results for the rumen microbial alpha diversity indices showed that different inositol doses significantly affected the Chao1, Observed_species, Shannon, Simpson, and Goods_coverage indices *in vitro* at 12 h (*p* < 0.05; Figure 2C) and the Shannon indices at 48 h (*p* < 0.01; Figure 2D). In the principal coordinate analysis (PCoA), clustering was distinct between different groups, and there was a clear tendency for dispersion between groups, suggesting that the addition of inositol affected the structure of the rumen microbial communities (Figures 2E,F).

### 3.3 Rumen metabolites *in vitro*

**Figure 3:**
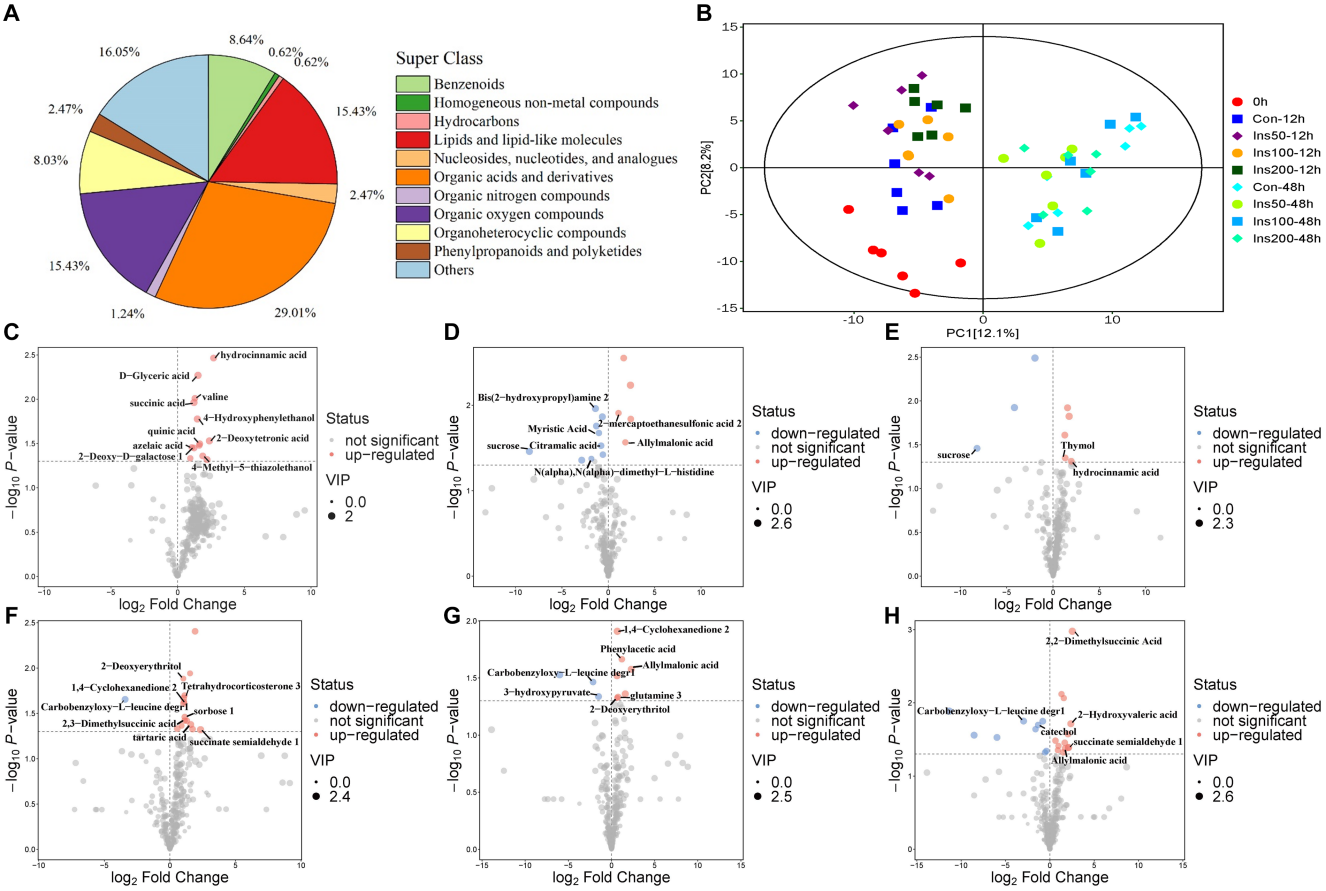
Rumen metabolites *in vitro*. Classification statistics of rumen metabolites *in vitro* **(A)**. Principal component analysis of rumen metabolites *in vitro* **(B)**. 0 h, rumen fluid not fermented *in vitro*. Con-12 h, basal diet. Ins50-12 h, Ins100-12 h, and Ins200-12 h represent basal diets supplemented with 50, 100, and 200 mg/kg DM inositol, respectively, fermented *in vitro* for 12 h. Con-48 h, basal diet. Ins50-48 h, Ins100-48 h, and Ins200-48 h represent basal diet supplemented with 50, 100, and 200 mg/kg DM inositol, respectively, fermented *in vitro* for 48 h. **(C–H)** Rumen differential metabolites in the *in vitro* experiment. Differential metabolites identified between control and **(C)** 50 mg/kg DM, **(D)** 100 mg/kg DM, and **(E)** 200 mg/kg DM groups at 12 h. Differential metabolites identified between control and **(F)** 50 mg/kg DM, **(G)** 100 mg/kg DM, and **(H)** 200 mg/kg DM groups at 48 h. Each dot represents a single metabolite. Red and blue scatter points represent significant upregulation and downregulation, respectively. Gray points represent no significant differences. Unnamed scatter points with significance represent substances that were not matched in the current database.

Rumen metabolite analysis was performed in the low-dose inositol groups at 12 and 48 h. A total of 162 metabolites were identified, of which organic acids and their derivatives accounted for the highest proportion (29.012%) of the total metabolites, lipids and lipid molecules; organic oxygen compounds; benzene ring compounds; polyketones of phenylpropane; nucleosides, nucleotides, and their analogs; organic nitrogen compounds; homogeneous non-metallic compounds; and hydrocarbons accounted for 15.432, 15.432, 8.642, 8.025, 2.469%; 1.235, 0.617, and 0.617%, respectively (Figure 3A).

Principal component analysis (PCA) was performed on all experimental groups. The first principal component (PC1) could explain 12.1% of the variance in the original data set. The PC1 results showed that groups with different fermentation times were separated, indicating that treatment time causes significant changes in metabolites in the samples. Samples in the different regions exhibited unique metabolic profiles. The second principal component explained 8.2% of the variance, and there was a clear separation between the control and the inositol addition groups at 12 h, indicating that the inositol addition treatment had an impact on the metabolites at this time point (Figure 3B).

Volcano plots were used to visualize the differential metabolite data between the inositol-supplemented and control groups at different fermentation times (Figures 3C–H). Multiple differential metabolite screening criteria, such as fold change, *p*-value, and variable importance in the projection (VIP) obtained using the OPLS-DA analysis model, were integrated and analyzed in multiple dimensions to obtain the most accurate differential metabolite analysis.

### 3.4 Rumen fermentation parameters and blood indices *in vivo*

Based on the results of the *in vitro* experiment, inositol doses of 0, 50, and 100 mg/kg DM were selected for the feeding experiment. The experimental results showed that different concentrations of inositol had no significant effect on volatile fatty acids (VFA) and NH_3_-N in the rumen, and none of the rumen fermentation parameters were affected by the interaction between inositol and experimental time points (*p* > 0.05; Supplementary Table S1).

The blood indices showed that there were no significant differences in total protein, albumin, globulin, total cholesterol, blood glucose, high-density lipoprotein, low-density lipoprotein, triglyceride, urea, free fatty acid, and β-hydroxybutyrate after inositol supplementation (*p* > 0.05). On day four, blood insulin levels in the inositol-supplemented group were significantly higher than those in the control (*p* < 0.05; *p* < 0.01; Table 3). Blood immune and oxidative stress indices showed no significant difference between the different doses of inositol, but IL-10, and superoxide varied after inositol supplementation and showed an increasing trend with the increasing days of treatment (Table 4). At each sampling time, blood HDL levels in the 50 mg/kg DM inositol group showed an increase compared to the control (Table 4). The addition of inositol increased glutathione peroxidase (GSH-Px) and decreased MDA (Table 4).

**Table 3:** Blood biochemical indicators at different levels of inositol supplementation on different experimental days^1^.

	Treatments (inositol mg/kg DM)										*p*-value		
Item	D1-I0	D1-I50	D1-I100	D4-I0	D4-I50	D4-I100	D7-I0	D7-I50	D7-I100	SEM	Ins	Day	I × D
TP, g/L	73.04	75.38	72.63	73.63	72.80	72.60	71.23	73.93	72.43	0.71	0.67	0.82	0.93
ALB, g/L	21.99	23.66	23.58	23.47	24.33	23.23	22.98	23.48	23.32	0.39	0.61	0.83	0.95
GLB, g/L	51.06	51.71	49.05	50.17	48.47	49.37	48.25	50.45	49.12	0.70	0.85	0.69	0.87
TC, mmol/L	1.68	1.85	1.63	1.76	1.94	1.61	1.62	1.74	1.60	0.06	0.37	0.79	0.99
GLU, mmol/L	3.28	3.23	3.38	2.95	3.13	3.07	3.06	3.18	3.14	0.07	0.84	0.36	0.98
HDL-C, mmol/L	0.92	0.97	0.85	0.95	1.02	0.83	0.88	0.96	0.89	0.04	0.40	0.97	0.99
LDL-C, mmol/L	0.45	0.56	0.46	0.53	0.54	0.46	0.47	0.46	0.46	0.02	0.56	0.69	0.87
TG, mmol/L	0.34	0.29	0.32	0.31	0.31	0.35	0.35	0.33	0.34	0.01	0.64	0.67	0.89
Urea, mmol/L	4.49	4.23	4.33	4.49	4.21	4.46	4.20	4.20	4.43	0.13	0.83	0.95	0.99
NEFA, mmol/L	0.13	0.33	0.24	0.22	0.28	0.15	0.21	0.18	0.17	0.02	0.33	0.77	0.51
BHBA, mmol/L	0.44	0.40	0.41	0.46	0.42	0.48	0.43	0.48	0.49	0.01	0.60	0.19	0.52
INS, μIU/mL	12.74	13.08	13.04	13.17^b^	14.92^a^	14.34^a^	14.04	13.77	15.18	0.22	0.21	<0.05	0.38

TP = total Protein; ALB = albumin; GLB = globulin; TC = total cholesterol; GLU = glucose; HDL-C = high density lipoprotein; LDL-C = low density lipoprotein; TG = triglyceride; NEFA = non-esterified fatty acid; BHBA = β- Hydroxybutyric acid; INS = insulin. SEM = standard error of mean; Ins = different inositol doses; Day = different sampling days; D × T = interaction of different inositol doses and different sampling days.

1 D1, 4, and 7 represent the first, fourth, and seventh days of the sampling period, respectively; I0, basal diet. I50, I100 represent basal diet supplemented with 50, 100 mg/kg DM inositol, respectively.

^a, b^ Means in the same sampling day with different superscripts are significantly different (*p* < 0.05).

**Table 4:** Blood immune and oxidative stress indicators at different levels of inositol supplementation on different experimental days^1^.

	Treatments (inositol mg/kg DM)										*p*-value		
Item	D1-I0	D1-I50	D1-I100	D4-I0	D4-I50	D4-I100	D7-I0	D7-I50	D7-I100	SEM	Ins	Day	I × D
IgA, μg/mL	244.48	242.07	242.26	251.21	244.48	242.70	255.92	244.86	245.40	2.31	0.41	0.62	0.98
IgG, mg/mL	75.12	74.83	76.19	72.28	79.84	78.82	79.40	79.79	83.18	0.96	0.26	0.06	0.62
IgM, μg/mL	2428.13	2550.51	2267.21	2553.66	2734.14	2697.90	2510.39	2616.23	2945.10	57.36	0.53	0.12	0.39
IL-2, pg./mL	1009.96	1027.10	1008.24	1094.32	1087.96	1016.86	1072.56	1135.95	1081.11	13.93	0.36	0.07	0.80
IL-4, pg./mL	54.48	51.89	51.44	48.77	47.13	52.93	49.36	53.86	52.64	1.60	0.92	0.74	0.88
IL-6, pg./mL	149.62	140.39	142.14	139.64	144.97	147.19	143.48	149.82	147.17	1.86	0.97	0.78	0.60
IL-10, pg./mL	141.61	138.38	140.50	152.74	146.78	150.30	151.33	153.54	155.34	1.70	0.79	<0.01	0.94
sCD14, ng/mL	21.65	20.75	21.17	23.15	21.11	21.31	20.29	21.27	21.55	0.24	0.54	0.32	0.19
TOS, U/mL	38.23	42.01	43.34	38.89	41.27	38.73	40.01	40.54	40.28	0.59	0.30	0.58	0.57
GSH-Px, pg./mL	1885.00	1948.50	1761.57	1716.87	1785.54	1769.20	1752.78	1927.63	1946.81	33.52	0.47	0.28	0.64
TAS, mmol/L	2.11	1.96	2.09	2.10	2.05	2.02	1.91	2.17	2.22	0.04	0.72	0.85	0.28
CAT, ng/mL	183.91	177.74	190.98	195.01	188.72	190.31	194.59	191.38	195.41	2.16	0.46	0.21	0.89
TNF-α, pg./mL	193.89	216.07	194.05	204.15	199.42	209.52	196.31	209.61	203.47	2.67	0.31	0.90	0.33
SOD, ng/mL	16.21	16.77	15.00	17.30	15.95	16.25	17.61	17.22	17.64	0.19	0.26	<0.01	0.20
MDA, nmol/mL	3.69	3.79	3.91	3.87	3.76	3.98	4.39	4.11	4.20	0.06	0.55	<0.01	0.74
T-AOC, U/mL	8.73	8.75	8.87	9.74	9.79	9.54	8.44	8.42	9.22	0.14	0.70	<0.01	0.65

IgA = immunoglobulin A; IgG = immunoglobulin G; IgM = immunoglobulin M; IL-2 = interleukin 2; IL-4 = interleukin 4; IL-6 = interleukin 6; IL-10 = interleukin 10; sCD14 = soluble cluster of differentiation14; TOS = total oxidant state; GSH-Px = glutathione peroxidase; TAS = total antioxidant status; CAT = catalase; TNF-α = tumor necrosis factor alpha; SOD = superoxide dismutase; MDA = malondialdehyde; T-AOC = total antioxidant capacity; SEM = standard error of mean; Ins = different inositol doses; Day = different sampling days; D × T = interaction of different inositol doses and different sampling days.

1 D1, 4, and 7 represent the first, fourth, and seventh days of the sampling period, respectively; I0, basal diet. I50, I100 represent basal diet supplemented with 50, 100 mg/kg DM inositol, respectively.

### 3.5 Microbial diversity in the rumen of sheep

**Figure 4:**
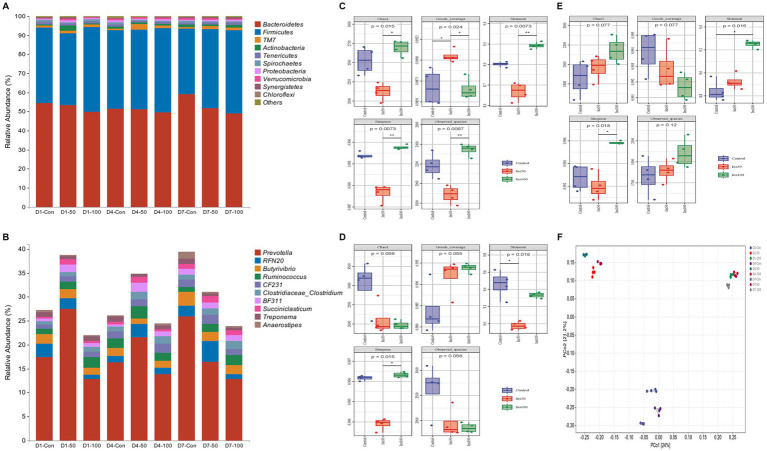
Effect of inositol supplementation on ruminal bacterial community. At the **(A)** phylum and **(B)** genus levels. D1-Con, basal diet. D1-50 and D1-100 represent basal diet supplemented with 50 and 100 mg/kg DM inositol, respectively, on the first day of sampling. D4-Con, basal diet. D4-50 and D4-100 represent basal diet supplemented with 50 and 100 mg/kg DM inositol, respectively, on the fourth day of sampling. D7-Con, basal diet. D7-50 and D7-100 represent basal diet supplemented with 50 and 100 mg/kg DM inositol, respectively, on the seventh day of sampling. The alpha diversity of the ruminal bacterial community. Estimated on the **(C)** first, **(D)** fourth, and **(E)** seventh day of the sampling period. **(F)** The beta diversity was estimated by principal coordinate analysis on the first, fourth, and seventh days of the sampling period.

A total of 3,102,488 original sequences were identified, and after removing low-quality sequences 2,690,978 valid sequences were obtained. After clustering and removing chimeras, a total of 2,513,273 high-quality sequences were analyzed. Bacteroidetes, Firmicutes, Actinobacteria, TM7, Tenericutes, Spirochaetes, Proteobacteria, and Verrucomicrobia were the most abundant phyla; and *Prevotella*, *RFN20*, *Butyrivibrio*, *Ruminococcus*, *CF231*, *BF311*, *Treponema*, *Clostridium*, and *Succiniclasticum* were the nine most abundant bacterial genera (Figures 4A,B).

The interaction between the inositol dose and the time of addition had significant effects on each phylum, except for Spirochaetes. The abundance of Bacteroidetes in the control was significantly higher than that in the 50 mg/kg DM (*p* < 0.05) and 100 mg/kg DM groups (*p* < 0.01). The abundance of Firmicutes in the 100 mg/kg DM group was significantly higher than that of the 50 mg/kg DM (*p* < 0.05) and control groups (*p* < 0.01). The abundance of Actinobacteria in the 50 mg/kg DM group was significantly higher than that of the 100 mg/kg DM (*p* < 0.05) and control groups (*p* < 0.01).

The abundance of *Prevotella*, *RFN20*, and *Succiniclasticum* in the 50 mg/kg DM group was significantly higher than that in the control (*p* < 0.05) and 100 mg/kg DM groups (*p* < 0.01). The abundance of *Butyrivibrio* in the 100 mg/kg DM group was significantly lower than in the other experimental groups (*p* < 0.05). The abundance of *Ruminococcus* in the 100 mg/kg DM group was significantly higher than that in the 50 mg/kg DM (*p* < 0.05) and control groups (*p* < 0.01). The abundance of *BF311* in the 50 mg/kg DM group was significantly higher than that in the 100 mg/kg DM (*p* < 0.05) and control groups (*p* < 0.01). *CF231*, *BF311*, *Treponema*, and *Succiniclasticum* had significantly lower abundances on day one than the other time points (*p* < 0.05). The abundance of *Clostridium* on day seven was significantly higher than that on day one (*p* < 0.05) and day four (*p* < 0.01).

Significant differences in alpha diversity were observed at different inositol concentrations, sampling times, and their interactions (Figures 4C–E). The Chao1 index of the 50 mg/kg DM group was significantly lower than that of the control group (*p* < 0.05) and was significantly higher on the first and fourth days of sampling compared to that on the seventh day (*p* < 0.05). The Shannon and Simpson indices in the 100 mg/kg DM group were significantly higher than those in the control (*p* < 0.05) and 50 mg/kg DM groups (*p* < 0.01).

The results of the beta diversity PCoA analysis showed that groups with the same inositol dose were clustered. PCoA1 explained 24% of the features, and groups with different doses of inositol addition had significant separation (Figure 4F). PCoA2 explained 21.2% of the features, and there was a significant separation between the control and other experimental groups, indicating differences in the microbial diversity between these groups.

### 3.6 Rumen metabolites of sheep

**Figure 5:**
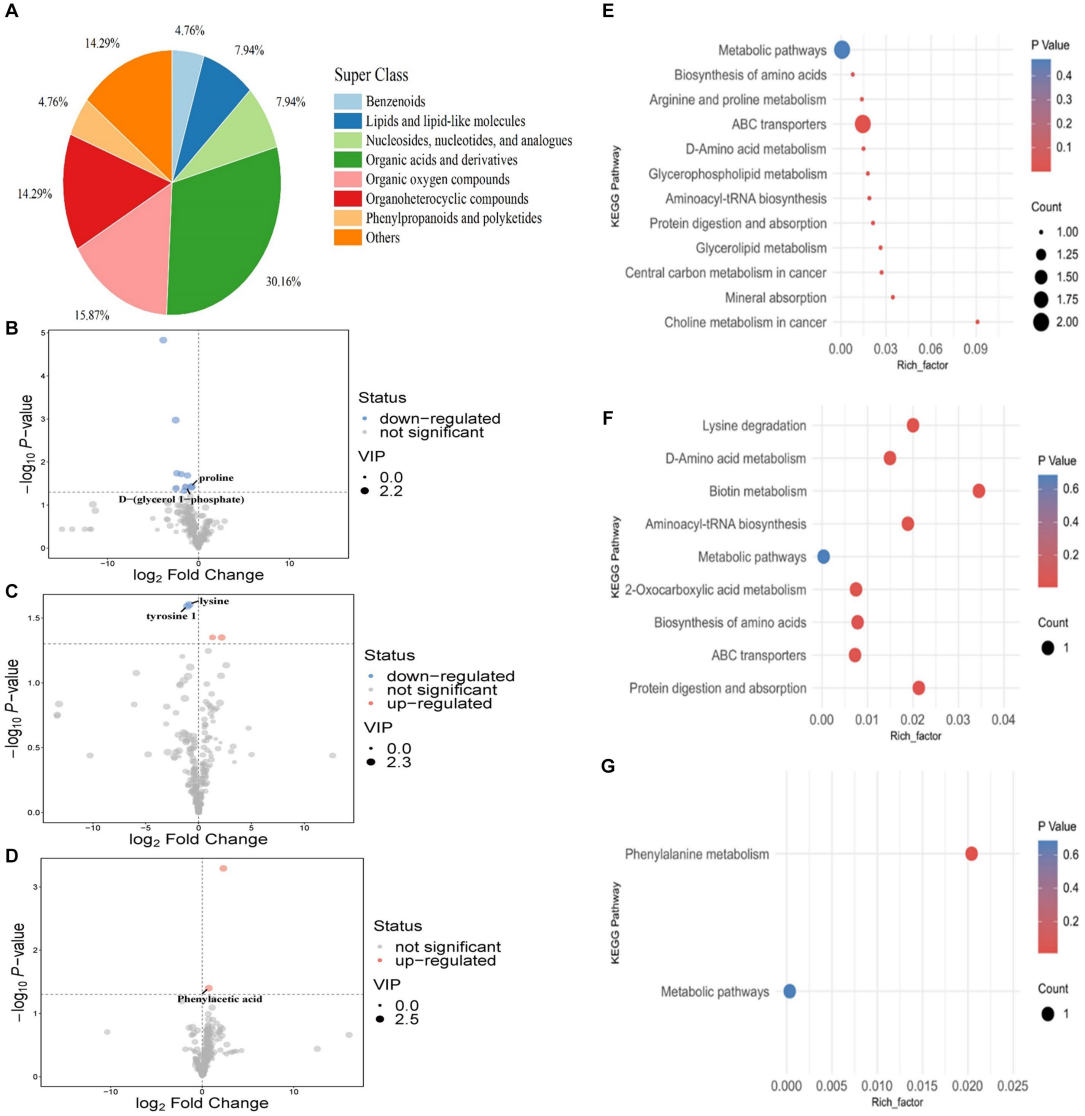
Rumen metabolites of sheep. Classification statistics of rumen metabolites **(A)**. Rumen differential metabolites after different lengths of inositol supplementation, first **(B)**, fourth **(C)**, and seventh **(D)** day of sampling. KEGG enrichment analysis of rumen differentiated metabolites in the feeding experiment **(E–G)**. Each point in the diagram represents a metabolic pathway; red and blue scatter points represent significant upregulation and downregulation, respectively. The horizontal coordinate where the points are located and the point size indicate the influence factor size of the path in topology analysis. The larger the size, the larger the influence factor.

A total of 63 metabolites were identified (Figure 5A). Among the secondary classifications in the HMDB (Human Metabolome Database), organic acids and their derivatives accounted for the highest proportion (30.159%) of the total metabolites.

On day one, the expression of proline and D-α-glycerol 1-phosphate was downregulated in the 50 mg/kg DM group compared to the control (Figure 5B). No differential metabolites were identified in the 100 mg/kg DM group. On the fourth day of inositol supplementation, lysine and L-tyrosine 1 were downregulated in the 100 mg/kg DM compared to the control group (Figure 5C). No differential metabolites were identified in the 50 mg/kg DM group. On day seven of inositol supplementation, the expression of phenylacetic acid in the 100 mg/kg DM group was upregulated compared to the control group (Figure 5D). No differential metabolites were identified in the 50 mg/kg DM group.

KEGG enrichment analysis of the differential metabolites showed that on the first day of inositol supplementation, the choline metabolism in the cancer pathway was most enriched in the 50 mg/kg DM vs. control comparison, and enrichment in the ABC transporter pathway was the most significant (Figure 5E). On the fourth day of inositol supplementation, the biotin metabolism pathway showed the highest enrichment and was the most significant in the 100 mg/kg DM vs. control comparison (Figure 5F). On the seventh day of inositol supplementation, only the phenylalanine metabolism pathway was enriched in the 100 mg/kg DM vs. control comparison (Figure 5G).

### 3.7 Blood metabolites

**Figure 6:**
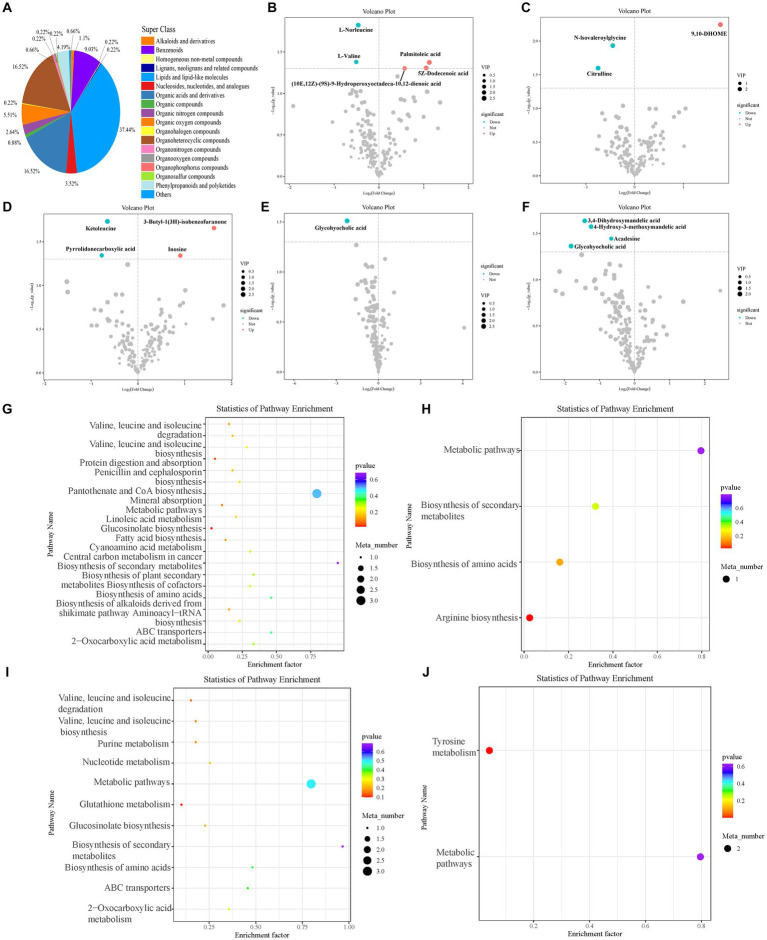
Blood metabolites of sheep. Classification statistics of blood metabolites **(A)**. Blood differential metabolites in the feeding experiment **(B–F)**. Differential metabolites between control and **(B)** 50 mg/kg DM and **(C)** 100 mg/kg DM groups on the first day of sampling. Differential metabolites between control and **(D)** 50 mg/kg DM groups on the fourth day of sampling. Differential metabolites between control and **(E)** 50 mg/kg DM and **(F)** 100 mg/kg DM groups on the seventh day of sampling. **(G–J)** KEGG enrichment analysis of blood differential metabolites in the feeding experiment. Enriched pathways in the control and **(G)** 50 mg/kg DM and **(H)** 100 mg/kg DM group comparisons on the first day of sampling. Enriched pathways in the control and **(I)** 50 mg/kg DM group comparison on the fourth day of sampling. Enriched pathways in the control and **(J)** 100 mg/kg DM group comparison on the seventh day of sampling.

A total of 454 blood metabolites were identified after sampling on days one, four, and seven. Among the secondary classifications in the HMDB database, lipids and lipid-like molecules constituted the highest proportions (Figure 6A).

On day one of sampling, 5Z-Dodecenoic acid (10E,12Z)-(9S)-9-hydroperoxyoctadeca-10,12-dienoic acid, and palmitoleic acid were significantly upregulated in the 50 mg/kg DM (Figure 6B) compared to the control group (*p* < 0.05), whereas 9,10-DHOME was significantly upregulated in the 100 mg/kg DM (Figure 6C) compared to the control group (*p* < 0.05). On day four of sampling, 3-Butyl-1(3H)-isobenzofuranone and inosine were significantly upregulated in the 50 mg/kg DM (Figure 6D) compared to the control group (*p* < 0.05). No significant differential metabolites were found on day four in the 100 mg/kg DM vs. control group comparison. On day seven of sampling, glycohyocholic acid was significantly downregulated in the 50 mg/kg DM (Figure 6E) compared to the control group (*p* < 0.05), and 4-Hydroxy-3-methoxymandelic acid, glycohyocholic acid, 3,4-Dihydroxymandelic acid, and acadesine were significantly downregulated in the 100 mg/kg DM (Figure 6F) compared with the control group (*p* < 0.05).

KEGG enrichment analysis of the blood differential metabolites showed that on day one of sampling, the 50 mg/kg DM group was enriched in the biosynthesis of secondary metabolites compared to the control group (Figure 6G), with the highest number of metabolites in metabolic pathways and the most significant enrichment in fatty acid biosynthesis. The 100 mg/kg DM group was mainly enriched in metabolic pathways compared to the control group (Figure 6H), with the most significant enrichment in arginine biosynthesis. On day four, the 50 mg/kg DM group was mainly enriched in the biosynthesis of secondary metabolites compared to the control group (Figure 6I), with the most significant enrichment in glutathione metabolism. No differential metabolites were identified in the 100 mg/kg DM group compared with the control. On day seven, no differential metabolic pathways were identified in the 50 mg/kg DM group compared to the control. The 100 mg/kg DM group was mainly enriched in metabolic pathways compared with the control and was significantly enriched in tyrosine metabolism (Figure 6J).

## 4 Discussion

The rumen is a natural fermenter in which a variety of rumen microorganisms live in symbiosis with the host and produce VFAs, microbial proteins, and vitamins for host growth and development (19). Rumen function is reflected by its fermentation parameters and is closely related to its microbial diversity. In this experiment, IVDMD, IVCPD, and NH_3_-N concentrations were higher in the low-dose than the high-dose inositol group. IVDMD and IVCPD reflect the simulated rumen utilization of feed *in vitro*, and the higher levels in the low-dose group indicate that a low dose of inositol in ruminant diets can promote the decomposition of feed carbohydrates and proteins by rumen microorganisms and the enrichment and colonization of the corresponding microbial community. NH_3_-N and free amino acids in the rumen are the primary precursors of microbial protein synthesis. A lower concentration of NH_3_-N affects the synthesis of microbial proteins, whereas a higher concentration leads to nitrogen loss. The higher concentration of NH_3_-N in the low-dose group may be due to an increase in the abundance of bacteria associated with protein decomposition, which converts feed proteins into oligopeptides and amino acids, with further degradation producing ammonia nitrogen. Gas production can be used to evaluate the ruminal fermentation efficiency and microbial activity. The 50 mg/kg DM group had the highest gas production, indicating that this dose is most efficient in regulating microbial activity in the rumen and promoting the fermentation of carbohydrates. VFAs, such as acetic acid, propionic acid, and butyric acid, are the main source of energy for ruminants and are the end products of feed carbohydrates. *In vitro*, the low-dose group had a higher concentration of TVFA at 3, 6, and 12 h. The concentrations of acetic acid, propionic acid, and butyric acid were significantly higher than those in the control and high-dose groups, and they also had a lower acetic acid/propionic acid (A/P) ratio. Acetic acid is produced by the degradation of cellulose and hemicellulose and is one of the main products of rumen microorganisms. It has the highest proportion in the rumen and can be used to synthesize milk fat together with butyric acid. Propionic acid can synthesize glucose, which is essential for the physiological activities of ruminants, by participating in the gluconeogenesis pathway and can be utilized by the mammary glands to produce lactose. The A/P ratio reflects the fermentation pattern of the rumen, and a decrease in this ratio indicates an increase in the efficiency of energy utilization in the animals’ diet. The 100 mg/kg DM dose significantly increased the production efficiency of valeric acid, a type of branched-chain volatile fatty acid; promoted the growth of fiber-degrading bacteria; and increased the efficiency of feed fiber degradation (20).

The rumen environment is a complex microbial system that is highly adaptive and competitive. Various microbial communities in the rumen produce enzymes involved in the breakdown of carbohydrates, nitrogenous compounds, and lipid compounds. Inositol addition influenced both the abundance and diversity of microorganisms in the rumen. Bacteroidetes and Firmicutes were the most abundant phyla, demonstrating the stability of the rumen’s internal environment. The bacteroidetes promote structural polysaccharide catabolism, and Firmicutes contain a large number of fiber-decomposing genera (21). *In vitro*, the level of Bacteroidetes in the inositol-supplemented group was significantly lower compared to the control at 48 h. This may be attributed to the higher accumulation of VFAs, resulting in a lower pH, leading to the death of some microorganisms intolerant of acid. Similar results were observed in the feeding experiment, where Bacteroidetes were significantly lower in abundance compared to the control group, whereas *Firmicutes* were all significantly higher than the control group. This suggests that in the inositol-supplemented group, the proportion of Firmicutes: Bacteroidetes increased relative to the control and that this represented an increase in the number of bacterial species that can ferment carbohydrates. Studies have shown that improving the ratio of Firmicutes: Bacteroidetes in the gut enables animals to absorb more energy from their diets (21).

Our results showed that the inositol supplementation increased the abundance of *Prevotella*, which is an important feed protein- and carbohydrate-degrading bacteria (22) that can improve branched-chain amino acid synthesis (23). Its abundance is affected by the composition of the diet and nutrition level (24) and positively correlates with dietary concentrate and protein levels (25). Inositol may promote the catabolic efficiency of feed proteins and enhance feed utilization in ruminants. Inositol increased the abundance of *Pseudomonadaceae_Pseudomonas in vitro*, a genus of uratolytic bacteria capable of hydrolysing urea to ammonia by producing urease, which is used for the synthesis of amino acids and microbial proteins (26). In the feeding experiment, inositol also increased the abundance of *RFN20*, *BF311*, *Treponema*, *Succiniclasticum*, and *Ruminococcus*. *RFN20* is a rumen-specific genus that is more closely associated with average daily gain in beef cattle (27). There are limited studies on the genus *BF311*, but its metabolic capacity may be similar to that of *Prevotella* spp. and it is involved in VFA-related metabolism (28). *Treponema* is a soluble fiber-degrading bacterium commonly found in the rumen that is capable of degrading plant polysaccharides (29). Succinic acid is an intermediate metabolite of the tricarboxylic acid cycle which can be produced or utilized by rumen microorganisms. *Succiniclasticum* is a Gram-negative genus that produces propionic acid by decarboxylation of succinate acid (30, 31) and is important in rumen microbial fermentation. *Ruminococcus* is an important intestinal commensal genus that helps the host degrade dietary phytopolysaccharides and has a great impact on both host health and the development of potential biological functions (32).

Inositol supplementation affected rumen metabolites, including those that regulate rumen function and maintain the health of its internal environment. Organic acids and their derivatives were the major metabolites detected. *In vitro*, the pathways of differential metabolites at 12 h were enriched in valine, leucine, and isoleucine metabolism and significantly enriched in the glycerol ester metabolism pathway. Inositol significantly upregulated the levels of succinic acid, hydrocinnamic acid, 4-Hydroxyphenylethanol, quinic acid, and azelaic acid metabolites. Studies have shown that 3-phenylpropionic acid and quinic acid inhibit bacterial virulence (33, 34), and azelaic acid can reduce intestinal inflammation (35). Inositol may improve the internal rumen environment by regulating these metabolites. Sucrose metabolism was significantly downregulated, suggesting that inositol may promote the efficiency of sucrose catabolism and enhance the efficiency of carbohydrate utilization in the rumen. After 48 h, the differential metabolic pathways were mainly enriched in the alanine, aspartic acid, and glutamic acid, and the differential metabolites were those that provide substrates for rumen microbial synthesis.

In the feeding experiment, 50 mg/kg DM inositol addition on day one resulted in the downregulation of proline and D-(glycerol 1-phosphate). Proline is an amino acid with complex biological functions, including cellular osmoregulation and protein synthesis. As a nutrient, it can promote the pathogenicity of different organisms, and disruption of proline metabolism can significantly attenuate the damage caused by some pathogens. For example, host restriction of nutrient supply is an important defense mechanism against *Brucella*, which is an important pathogenic bacterium in ruminants that requires proline to be supplied by the host for its activity (36). Inhibition of proline metabolism may be an effective strategy for the treatment of damage caused by some pathogens (37). The tumor-suppressive effect of inositol and its derivatives may be due to inhibition of the ERK–MAPK or P13K–Akt pathways (38), but the validation of the ability of inositol addition to modulate the levels of choline compounds is still lacking. On the fourth day of inositol supplementation, lysine and tyrosine expression were downregulated in the 100 mg/kg DM inositol group compared to the control; mass spectrometry results from previous experiments indicate that organophosphorus agents can form covalent bonds with lysines and tyrosines in proteins without a serine active site (39), inositol is metabolized in animals to form inositol phosphates, and further experiments are required to investigate whether inositol binds these two amino acids during metabolism. On day seven of inositol supplementation, phenylacetic acid metabolism was significantly upregulated in the phenylalanine metabolic pathway in the 100 mg/kg DM inositol group compared to the control. Phenylacetic acid is a metabolite of phenylbutyric acid that is further metabolized to phenylacylglutamine and phenylbutyrylglutamine, which are excreted through the urine (40). Phenylacetic acid modulates endoplasmic reticulum chemical stress and plays a protective role in a variety of diseases (41). In rumen microorganisms, phenylacetic acid can be formed by the conversion of phenylalanine (42) and exerts a stimulatory effect on the fiber digestion of *Ruminococcus* (43). The rumen fluid metabolite results indicated that inositol addition produced some variation in sheep rumen metabolites and may improve animal health.

The addition of inositol only had a small effect on the blood biochemical indices, but there were some clear trends. The 50 mg/kg DM inositol supplementation promoted an increase in total blood cholesterol levels, and an upregulation of blood high-density lipoprotein was observed with increasing treatment time. Cholesterol is an important regulatory molecule in the body, and its excess or deficiency leads to the development of diseases. It can be distributed in various parts of the body as a plasma protein carrier of the fat-soluble vitamins K and E and can be used as a carrier for cellular drugs. Regulation of cholesterol levels is of great importance to animal health. High-density lipoproteins remove excess cholesterol and prevent diseases caused by high cholesterol levels, such as atherosclerosis (44). In previous studies, the addition of inositol reduced cholesterol levels (45), which differs from the increase observed after the addition of inositol in this study, which may be related to the increase in HDL levels. On the fourth day of inositol supplementation, the insulin level was significantly higher than that of the control group. Insulin is the only hormone that can reduce blood glucose concentration, and an increase in its synthesis can reduce the blood glucose concentration in animals after feeding, increase feed intake, and promote nutrient deposition, which is beneficial to the growth and development of animals (46).

Increasing inositol supplementation time increased the levels of IgG, IgM, and IL-4. IL-4 can promote B cell differentiation into antibody-secreting cells and enhance humoral immunity (47), and IgG and IgM can mediate humoral immune responses. An increase in the levels of both can promote specific immune functions, and the continuous addition of inositol may enhance the immune ability of animals. The blood GSH-Px level significantly increased with increasing treatment time, and the MDA levels decreased. GSH-Px is an important cellular antioxidant enzyme that removes free radicals (48). MDA is one of the final products of the peroxidation of polyunsaturated fatty acids in cells, and an increase in free radicals in cells increases the level of MDA (49); therefore, the level of MDA is used as a marker for oxidative stress and antioxidant status in animals. Changes in these levels after inositol supplementation may indicate the potential antioxidant capacity of inositol in ruminant nutrition. Current studies on the antioxidant capacity of inositol, focusing on aquatic animals, have shown that inositol can improve antioxidant and immune capacities (13, 50, 51). However, there are few studies on inositol in ruminants, and the mechanism by which inositol affects their immunity and antioxidant activity requires further investigation.

The addition of inositol promoted the production of lipid or lipid-like molecules, such as palmitoleic acid and the nucleotide metabolite inosine, and inhibited the metabolism of glycohyocholic acid. Palmitoleic acid is capable of regulating different metabolic processes, and studies have shown that phospholipid levels of palmitoleic acid in the blood are associated with elevated high-density lipoproteins (52). Palmitoleic acid inhibits steatosis in the liver and improves insulin sensitivity by modulating GLUT-4 and AMPK phosphorylation (53). Downregulation of SIRT3 gene expression by palmitoleic acid regulates the gluconeogenesis pathway and maintains normal blood sugar levels in the body (54). Palmitoleic acid can also inhibit inflammation and reduce systolic blood pressure to prevent hypertensive diseases (55), thereby playing an important role in maintaining the health of the body.

Inosine is an important secondary metabolite of purine metabolism that participates in a variety of biochemical processes. Intracellularly, it can act as a molecular messenger to participate in physiological activities related to cellular signaling pathways, as well as having immunomodulatory and neuroprotective effects. Extracellularly, inosine can inhibit the production of pro-inflammatory factors, such as TNF-α and IL-1, and slow down the macrophage-mediated endotoxin inflammatory response (56), blocking the production of superoxide induced by formyl peptides (57). The exogenous addition of inosine also mitigates cellular damage and protects body tissues (58), and an increase in the metabolic level of inosine indirectly enhances the immune capacity of the body.

Glycohyocholic acid is the glycine-bound form of primary bile acid and has been implicated in the regulation of intestinal flora activity (59). Bile acids are synthesized from cholesterol by hepatocytes and are involved in many biological functions. Free bile acids in the blood can be removed by transporter proteins located on the basement membrane of hepatocytes (60). Blood concentrations of bile acids are elevated in patients with non-alcoholic fatty liver disease (NAFLD) who exhibit insulin resistance (61), and inositol, with its insulin-mimetic properties, can play a role in diseases associated with insulin resistance and has the potential to modulate NAFLD (62). Glycohyocholic acid metabolism was significantly downregulated in the inositol-supplemented group compared to the control, suggesting that inositol is involved in lipid metabolism and regulates the level of sterols in ruminants.

There are still some shortcomings in this study. The gas composition in total gas production, especially CH_4_, was not analyzed in the *in vitro* experiment. Further classification of the gas produced by fermentation can further explore the effects of different inositol dosages on rumen nutrient digestion, microbial diversity, and environmental pollution of sheep. The role of inositol in the growth performance of sheep should be further studied in the future, and the mechanism of inositol’s influence on sheep’s bodies should be discussed in more detail at the molecular level according to the results obtained in the experiment.

## 5 Conclusion

Inositol was found to improve rumen fermentation and affect its microbial diversity and metabolism *in vitro*. These results were validated in the feeding experiment, indicating the potential of inositol to reduce inflammation and improve animal health.

## Data availability statement

The datasets presented in this study can be found in online repositories. The names of the repository/repositories and accession number(s) can be found here: NCBI sequence Read Archive (SRA), accession numbers PRJNA1027573 and PRJNA1027802.

## Ethics statement

The animal study was approved by all experimental procedures were performed in accordance with the Guidelines for the Care and Use of Experimental Animals of the Jilin Agricultural University (JLAU-ACUC2022-006). The study was conducted in accordance with the local legislation and institutional requirements.

## Author contributions

GY: Writing – original draft, Data curation, Software, Validation, Visualization. ZS: Writing – review & editing, Resources. ZW: Writing – review & editing, Investigation. YX: Writing – review & editing, Investigation. LC: Writing – review & editing, Investigation. GQ: Writing – review & editing, Formal analysis, Methodology. NA: Writing – review & editing, Investigation. HL: Writing – review & editing, Supervision. XZ: Writing – review & editing, Formal analysis, Methodology. QW: Writing – review & editing, Investigation. WZhan: Writing – review & editing, Supervision. WZhao: Writing – review & editing, Supervision. TW: Writing – review & editing, Funding acquisition, Supervision. YZ: Writing – review & editing, Formal analysis, Methodology.
