# Supplementary material for: Mechanistic insights into inositol-mediated rumen function promotion and metabolic alteration using in vitro and in vivo models
Source: Front Vet Sci. 2024 Feb 16;11:1359234. doi: 10.3389/fvets.2024.1359234 (PMC10904589; doi:10.3389/fvets.2024.1359234)
Supplement: Supplementary file 1 [file Table_1.DOCX]

Supplementary Material

# Supplementary Figures and Tables

1. Table S1. Rumen fermentation parameters at different levels of inositol addition on different experimental days ^1^

|  |  | Treatments（Inositol mg/kg DM） | | | | | | | | |  | *P*-value | | |
| --- | --- | --- | --- | --- | --- | --- | --- | --- | --- | --- | --- | --- | --- | --- |
| Item | Time(h) | D1-I0 | D1-I50 | D1-I100 | D4-I0 | D4-I50 | D4-I100 | D7-I0 | D7-I50 | D7-I100 | SEM | Ins | Day | I×D |
| pH value | 0 | 6.69 | 6.86 | 7.07 | 6.57 | 6.66 | 6.79 | 6.59 | 6.59 | 6.87 | 0.05 | 0.06 | 0.20 | 0.97 |
|  | 2 | 6.39 | 6.19 | 6.55 | 6.39 | 6.24 | 6.41 | 6.27 | 6.21 | 6.45 | 0.05 | 0.12 | 0.87 | 0.96 |
|  | 4 | 6.23 | 6.13 | 6.48 | 6.22 | 6.08 | 6.24 | 6.12 | 6.14 | 6.41 | 0.05 | 0.07 | 0.68 | 0.86 |
|  | 8 | 6.62 | 6.57 | 6.69 | 6.39^ab^ | 6.24^b^ | 6.63^a^ | 6.34 | 6.38 | 6.55 | 0.04 | 0.07 | 0.08 | 0.80 |
|  | 12 | 6.56 | 6.52 | 6.67 | 6.44 | 6.46 | 6.78 | 6.54 | 6.71 | 6.88 | 0.04 | <0.05 | 0.26 | 0.78 |
|  |  |  |  |  |  |  |  |  |  |  |  |  |  |  |
| TVFA  (mmol/L) | 0 | 58.16 | 60.13 | 61.28 | 67.93 | 77.42 | 75.76 | 59.33 | 62.59 | 58.96 | 2.62 | 0.74 | 0.06 | 0.98 |
|  | 2 | 78.62 | 66.18 | 73.40 | 81.61 | 85.30 | 72.79 | 64.31 | 65.73 | 70.54 | 3.80 | 0.98 | 0.07 | 0.76 |
|  | 4 | 64.24 | 76.76 | 71.70 | 74.01 | 74.47 | 88.91 | 67.26 | 91.66 | 79.17 | 5.86 | 0.83 | 0.15 | 0.93 |
|  | 8 | 54.11 | 61.23 | 55.24 | 45.13^b^ | 63.50^a^ | 57.23^a^ | 62.55 | 65.35 | 54.67 | 6.64 | 0.61 | 0.12 | 0.10 |
|  | 12 | 46.95 | 58.12 | 45.60 | 45.23 | 56.93 | 55.62 | 53.54 | 53.86 | 55.57 | 2.59 | 0.48 | 0.76 | 0.67 |
|  |  |  |  |  |  |  |  |  |  |  |  |  |  |  |
| Acetate acid  (mmol/L) | 0 | 35.08 | 40.05 | 41.91 | 44.22 | 50.68 | 48.92 | 37.66 | 40.81 | 40.83 | 1.51 | <0.05 | 0.31 | 0.99 |
|  | 2 | 48.64 | 42.32 | 45.90 | 52.37 | 54.66 | 45.60 | 40.37 | 41.21 | 44.87 | 2.28 | 0.96 | <0.05 | 0.73 |
|  | 4 | 43.83 | 43.24 | 38.04 | 48.67 | 47.63 | 55.39 | 43.31 | 57.91 | 50.38 | 3.64 | 0.61 | <0.05 | 0.75 |
|  | 8 | 35.19 | 39.90 | 36.01 | 30.47^b^ | 42.64^a^ | 36.96^ab^ | 36.35 | 42.08 | 35.63 | 4.00 | 0.71 | 0.09 | 0.10 |
|  | 12 | 30.84 | 38.44 | 29.59 | 30.77 | 38.71 | 36.78 | 35.56 | 35.43 | 36.44 | 1.66 | 0.48 | 0.64 | 0.65 |
|  |  |  |  |  |  |  |  |  |  |  |  |  |  |  |
| Propionate acid  (mmol/L) | 0 | 12.45 | 9.60 | 13.58 | 12.22 | 14.08 | 13.87 | 11.44 | 11.35 | 10.02 | 0.66 | 0.89 | 0.33 | 0.62 |
|  | 2 | 18.20 | 13.95 | 15.66 | 17.03 | 17.69 | 15.67 | 14.07 | 13.74 | 13.98 | 1.02 | 0.99 | 0.17 | 0.81 |
|  | 4 | 16.57 | 12.59 | 11.93 | 14.26 | 14.95 | 19.20 | 14.52 | 19.51 | 15.75 | 1.46 | 0.96 | 0.32 | 0.82 |
|  | 8 | 11.11 | 12.45 | 10.20 | 8.44^b^ | 12.07^a^ | 11.10^ab^ | 10.12 | 13.28 | 10.39 | 1.74 | 0.62 | 0.12 | 0.16 |
|  | 12 | 8.04 | 10.80 | 8.90 | 8.46 | 10.46 | 10.51 | 11.00 | 10.21 | 10.13 | 0.65 | 0.70 | 0.77 | 0.78 |
|  |  |  |  |  |  |  |  |  |  |  |  |  |  |  |
| Isobutyric acid  (mmol/L) | 0 | 1.09 | 0.88 | 1.10 | 0.91 | 0.89 | 0.97 | 0.74 | 0.70 | 0.69 | 0.04 | 0.45 | <0.01 | 0.80 |
|  | 2 | 0.76 | 0.70 | 0.75 | 0.79 | 0.78 | 0.72 | 0.57 | 0.59 | 0.72 | 0.03 | 0.85 | 0.08 | 0.81 |
|  | 4 | 0.63 | 0.73 | 0.57 | 0.64 | 0.69 | 0.79 | 0.58 | 0.73 | 0.70 | 0.09 | 0.57 | 0.92 | 0.76 |
|  | 8 | 0.53 | 0.68 | 0.61 | 0.49 | 0.59 | 0.56 | 0.53 | 0.58 | 0.55 | 0.05 | 0.91 | <0.05 | 0.28 |
|  | 12 | 0.53 | 0.69 | 0.53 | 0.52 | 0.56 | 0.58 | 0.56 | 0.59 | 0.62 | 0.02 | 0.70 | 0.77 | 0.78 |
|  |  |  |  |  |  |  |  |  |  |  |  |  |  |  |
| Butyric acid  (mmol/L) | 0 | 7.62 | 7.45 | 8.81 | 7.94 | 9.32 | 9.26 | 7.09 | 7.77 | 5.59 | 0.54 | 0.90 | 0.34 | 0.83 |
|  | 2 | 8.56 | 7.16 | 8.94 | 9.06 | 9.66 | 8.64 | 7.55 | 8.30 | 8.85 | 0.53 | 0.79 | 0.28 | 0.87 |
|  | 4 | 8.20 | 7.93 | 5.39 | 8.52 | 9.22 | 11.13 | 7.09 | 11.31 | 10.33 | 2.09 | 0.46 | 0.96 | 0.77 |
|  | 8 | 5.71 | 6.51 | 5.83 | 4.57^b^ | 6.72^ab^ | 7.13^a^ | 6.30 | 7.85 | 6.66 | 0.76 | 0.88 | 0.21 | 0.07 |
|  | 12 | 4.96 | 6.37 | 5.27 | 4.27 | 5.89 | 6.30 | 5.06 | 6.22 | 6.93 | 0.37 | 0.15 | 0.86 | 0.79 |
|  |  |  |  |  |  |  |  |  |  |  |  |  |  |  |
| Isovaleric acid  (mmol/L) | 0 | 1.89 | 1.49 | 1.84 | 1.59 | 1.45 | 1.64 | 1.37 | 1.15 | 1.17 | 0.08 | 0.40 | <0.05 | 0.95 |
|  | 2 | 1.24 | 1.08 | 1.09 | 1.13 | 1.19 | 1.07 | 0.85 | 0.92 | 1.08 | 0.06 | 0.98 | 0.28 | 0.81 |
|  | 4 | 0.96 | 1.00 | 0.86 | 0.94 | 0.97 | 1.14 | 0.82 | 1.04 | 1.01 | 0.12 | 0.66 | 0.91 | 0.78 |
|  | 8 | 0.85 | 0.95 | 0.91 | 0.71 | 0.88 | 0.83 | 0.77 | 0.85 | 0.84 | 0.08 | 0.85 | <0.05 | 0.29 |
|  | 12 | 0.83 | 1.18 | 0.89 | 0.78 | 0.82 | 0.89 | 0.83 | 0.88 | 0.92 | 0.04 | 0.48 | 0.76 | 0.55 |
|  |  |  |  |  |  |  |  |  |  |  |  |  |  |  |
| Valerate acid  (mmol/L) | 0 | 0.88 | 0.66 | 1.03 | 1.06 | 1.01 | 1.11 | 1.03 | 0.80 | 0.66 | 0.07 | 0.66 | 0.40 | 0.74 |
|  | 2 | 1.23 | 0.97 | 1.07 | 1.24 | 1.32 | 1.11 | 0.89 | 0.97 | 1.05 | 0.06 | 0.98 | <0.05 | 0.67 |
|  | 4 | 1.17 | 0.94 | 0.82 | 0.98 | 1.03 | 1.26 | 0.95 | 1.17 | 1.01 | 0.19 | 0.59 | 0.87 | 0.72 |
|  | 8 | 1.80 | 1.18 | 0.82 | 0.46^b^ | 0.61^ab^ | 0.66^a^ | 0.56 | 0.72 | 0.60 | 0.10 | 0.68 | <0.05 | 0.15 |
|  | 12 | 0.47 | 0.65 | 0.51 | 0.44 | 0.50 | 0.58 | 0.53 | 0.52 | 0.54 | 0.03 | 0.48 | 0.98 | 0.63 |
| A/P ratio | 0 | 3.49 | 4.19 | 3.68 | 3.87 | 3.78 | 3.69 | 3.69 | 3.71 | 4.32 | 0.12 | 0.73 | 0.89 | 0.51 |
|  | 2 | 2.93 | 3.35 | 3.10 | 3.22 | 3.13 | 3.05 | 2.96 | 3.08 | 3.29 | 0.08 | 0.77 | 0.99 | 0.75 |
|  | 4 | 2.97 | 3.61 | 3.31 | 3.47 | 3.27 | 2.96 | 3.09 | 3.08 | 3.37 | 0.11 | 0.48 | 0.83 | 0.38 |
|  | 8 | 3.42 | 3.58 | 3.34 | 3.71 | 3.59 | 3.50 | 3.66 | 3.36 | 3.63 | 0.10 | 1.00 | 0.80 | 0.90 |
|  | 12 | 3.74 | 3.00 | 3.24 | 3.75 | 3.75 | 3.63 | 3.81 | 3.64 | 3.81 | 0.12 | 0.62 | 0.37 | 0.90 |
|  |  |  |  |  |  |  |  |  |  |  |  |  |  |  |
| NH_3_-N (mg/dL) | 0 | 28.50 | 26.38 | 32.00 | 22.01 | 22.39 | 26.18 | 28.60 | 26.98 | 30.36 | 1.38 | 0.46 | 0.23 | 1.00 |
|  | 2 | 32.75 | 25.49 | 29.63 | 26.17 | 25.44 | 27.03 | 21.82 | 31.15 | 25.80 | 1.43 | 0.99 | 0.63 | 0.47 |
|  | 4 | 23.10 | 22.99 | 22.90 | 22.01 | 21.23 | 23.46 | 22.43 | 21.41 | 24.58 | 1.04 | 0.81 | 0.96 | 0.99 |
|  | 8 | 21.96 | 17.17 | 23.24 | 16.68 | 21.72 | 23.24 | 17.28 | 16.46 | 17.81 | 1.11 | 0.49 | 0.36 | 0.65 |
|  | 12 | 17.40 | 17.92 | 17.68 | 20.74 | 17.41 | 18.85 | 18.09 | 16.07 | 16.70 | 0.86 | 0.77 | 0.66 | 0.97 |

SEM = standard error of mean; Ins = different inositol doses; Day = different sampling days; D×T = interaction of different inositol doses and different sampling days.

^1^ D1, 4, and 7 represent the first, fourth, and seventh days of the sampling period, respectively; I0, basal diet. I50, I100represent basal diet supplemented with 50, 100 mg/kg DM inositol, respectively.
